# Contributory factors related to patient safety incidence: A nursing perspective

**DOI:** 10.4102/hsag.v29i0.2296

**Published:** 2024-05-06

**Authors:** Gifty Adu, Sibusiso M. Zuma

**Affiliations:** 1 Department of Health Studies, School of Social Sciences, University of South Africa, Pretoria, South Africa

**Keywords:** contributory factors, healthcare, knowledge, patient safety, professional nurse, safety practices

## Abstract

**Background:**

There are growing concerns about patient safety and quality assurance enhancement in the healthcare setting because of the increase in the incidence of patient harm and adverse events over the years.

**Aim:**

This study explored the contributory factors associated with patient safety practices.

**Setting:**

The study was conducted in two private hospitals in Gauteng province, South Africa.

**Methods:**

A qualitative approach was used to gain an in-depth understanding of the issues pertaining to patient safety incidence. Purposive sampling was used to select professional nurses practicing within the two private hospitals. Thematic analysis was used. The study utilised the Donabedian model of patient safety and quality.

**Results:**

The study revealed that majority of the professional nurses did not understand the concept of patient safety; there was poor communication between the multidisciplinary team. There was poor adherence to patient safety policies.

**Conclusion:**

Patient safety issues remain an issue of concern in public health. There is a need for nurses to be capacitated on the implementation of patient safety programmes as well as improving communication within the multidisciplinary team. Identifying and addressing risk and contributory factors will help reduce the global burden of patient harm.

**Contribution:**

The study has presented the challenges as seen with patient safety and made recommendations on how to improve patient safety from the nursing perspective. It is anticipated that the results of this study may be used to create awareness on patient safety issues. This should promote a good healthcare climate in private healthcare institutions.

## Introduction

Patient safety was brought to light by the landmark report published in 1999 by the Institute of Medicine (IOM) in the United States. The report, entitled ‘To Err is Human: Building a Safer Health System’, highlighted how vital it is that patient safety forms an important fundamental concern on the agendas of all nations (Cho & Choi 2018; Mortell 2019; WHO 2017).

Globally, for every 10 patients receiving care in a primary and outpatient healthcare, four are harmed, and 80% of such incidents are preventable. One hundred and thirty-four million incidents of patient harm occur annually because of hazardous care in hospitals in both low- and middle-income countries. This contributes to 2.6 million deaths annually. One of the 10 leading causes of death is patient harm resulting from poor care and one in 10 patients incurs harm while assessing healthcare, of which 50% is avoidable (WHO 2017). Eight million of the deaths that occur in low- and middle-income countries are attributed to poor quality healthcare (WHO 2017). An investment in decreasing patient harm will lead to substantial economic savings and improved patient outcomes.

Nursing plays an important role in transforming the healthcare environment for all stakeholders (WHO 2020). The core of nursing is the rendering of quality care to improve patient health outcomes. The universal nursing labour force is approximately 27.9 million of which 19.3 million are professional nurses (WHO 2020). Nurses play a vital role in guaranteeing patient safety by observing patients for clinical deterioration, identifying faults and near misses, having an insight into the care process and flaws embedded in certain structures and carrying out various activities to certify that high-quality care is received by patients (WHO 2020).

The International Council of Nurses (ICN), an alliance of more than 130 national nurses’ associations, acknowledges that although healthcare interventions are aimed at benefiting both the patients and the public, there is nevertheless an element of risk that errors, and adverse events will occur because of the multifaceted combination of processes, machineries and human features related to healthcare (Barkhordari-Sharifabad & Mirjalili 2020; Twigg et al. 2021). However, there is a rising indication that insufficient institutional staffing levels are associated with increased patient safety incidents, including adverse events such as ‘patient falls, bedsores, medication errors, nosocomial infections, and other avoidable patient harm’ (Liu & Aungsuroch 2019:1445). Accordingly, the ICN believes that the heightening of patient safety comprises an extensive choice of activities in the enrolment, teaching and retention of healthcare professionals, performance upgrading, risk management and environmental safety, including safe use of medicine, infection control and safe clinical practice (Barkhordari-Sharifabad & Mirjalili 2020).

As a country, South Africa faces a plethora of problems related to healthcare quality in both the private and public health sectors (Mayeng & Wolvaardt 2015). Some of the quality problems that have been identified include avoidable errors such as medication error, pressure ulcers, lack of resources, inadequate diagnoses and treatment, drug shortages, inefficient use of resources and poor delivery system (Mayeng & Wolvaardt 2015).

Despite the various policies developed and approved by both the WHO and the South African National Department of Health to improve patient safety and decrease patient safety incidents, patient safety remains an area of concern with an increasing number of negative outcomes. Moreover, the majority of patient safety issues involve nurses. As discussed by Stellenberg (2018:1–3),negative patient safety incidents lead to poor patient health outcomes, as these often prolong the patients’ hospital stay, increase the patients’ hospital costs and decrease the trust between patients and healthcare givers.

A study conducted by Kubheka et al. (2019) to explore the interplay between health system, patient safety culture and second victimhood showed that healthcare professionals may be said to be the second victims of patient safety incidents as they experience the following phases, namely, error involvement, guilt, frustrations, burnout, depression, diminishing empathy and fear, when patient safety incidents occur. This has an adverse impact on the quality of care delivered and patient health outcomes.

Various studies have overlapped identifying key elements that play a role in patient safety. These include the following: technology, nursing workload, working conditions, education and training, appropriate knowledge and skill mix, patient safety culture (incident reporting, communication, team building) and organisational climate (Amaniyan et al. 2019; Begg et al. 2018; Jang, Lee & Lee 2022; Lee et al. 2021; WHO 2020).

Observations in the health sector provide evidence of patient harm through the increased prevalence of surgical complications, patient deaths as a result of clinical procedures and the increased incidence of healthcare-associated infections. Furthermore, it would appear that there are limited capacity-building and preventative initiatives to reduce patient harm while under the care of health professionals, including nurses. This could be attributed, but not limited, to both inadequate knowledge and a lack of guidelines to promote patient safety practices in the healthcare establishments. There is a need to assess the knowledge and practices of professional nurses who are the frontline managers in the healthcare setting and also to develop guidelines to improve their adherence to patient safety policies.

### Research objectives

This study aims to describe the contributory factors associated with patient safety practices and make recommendations to improve patient safety practices.

### Framework

**FIGURE 1:**
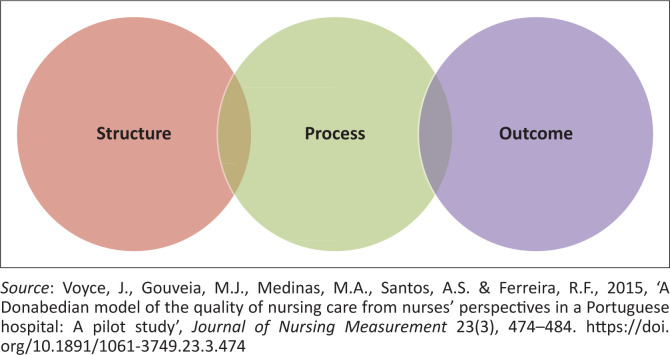
The Donabedian model of patient safety and quality.

This study investigated patient safety from the perspective of nurses who are employed in private healthcare establishments. In their practice, nurses follow the scientific process in providing healthcare and evaluating the outcomes. Accordingly, as depicted in Figure 1, the Donabedian model of patient safety and quality was deemed appropriate for the purposes of this study because the data-collection tool utilised the nursing process approach to gather the requisite data, evaluate the participants’ responses and also investigate the contributing factors as mentioned in the theoretical framework.

The Donabedian model assumes the existence of three essential factors in assessing quality: namely, structure, process and outcome (Voyce et al. 2015).

*Structure* denotes the setting within which medical care is delivered and the instrumentalities of each product, including the attributes of the system, the health care service provider and/or the patient (Voyce et al. 2015).

*Process* denotes the established actions that take place between healthcare givers and also between healthcare givers and patients, which include technical and interactive relations (Voyce et al. 2015).

*Outcome* denotes the shortcomings for the health and well-being of persons and the public, which includes clinical consequences, quality of life and gratification with the healthcare delivered (Voyce et al. 2015).

These three factors must correlate in the same direction to guarantee patient safety and progressive patient care outcomes. This framework indicates that the setting within which care is delivered may affect the care-giving process, thereby either improving or reducing the quality of such care. Both the structure and the process have an impact on the outcomes, thus a change in structure affects the process while a change in the process may in turn have an impact on the patients’ health outcomes.

## Research methods and design

### Study design

A qualitative exploratory contextual methodology was utilised to explore the contributory factors associated with patient safety incidences among professional nurses. The qualitative design was adopted as it is flexible, it can generate a wide range of ideas and opinions that individuals harbour with regard to the issue under study, and it also allowed an in-depth exploration of respondent’s attitude, experiences and intentions (Creswell & Creswell 2018).

### Study setting

This study was conducted at two private healthcare settings in Gauteng. These hospitals have both in-and-out patient departments. The hospitals selected belong to a large private healthcare group with a high patient and nurse populations as this would fulfil the study’s purpose and enhance the study outcomes.

### Study population and sampling strategy

The population under study comprised professional nurses working within the private healthcare sector in Gauteng province, South Africa. The accessible population targeted for this study was approximately 380. Purposive sampling was utilised to target and identify professional nurses who had experience in clinical nursing and were involved in patient care.

### Data collection

An open guide was used to collect data from individuals on the current prevailing practices of nurses within the private healthcare setting. Participants filled in a questionnaire (open guides) in the presence of the researcher. The questionnaire focussed on contributory factors with regard to patient safety practices and incidents. Prior to data collection, the questionnaire (open guide) was piloted using nurses from another private hospital with characteristics similar to the population under study. The data were collected over a period of 2 months because of the nature of shift work done by the nurses (November and December 2020). An average of 45 min was used by each participant to complete the guide using the English Language.

### Data analysis

The responses were entered into the software ATLAS.ti to generate themes. The themes and sub-themes, where applicable, are presented and supported by direct quotes from participants.

### Trustworthiness

Credibility: An orientation training session was conducted with the participants prior to the data collection to clarify and explain the questions contained in the guide. The data collected were shared with participants to confirm the accuracy and facilitate member checking.

Dependability: All the material emanating from this study was reviewed by the researcher’s supervisor. In addition, an independent co-coder and a statistician reviewed and had input into the study design, research method, data collection and analysis of the results to enhance the dependability of the study.

Conformability: Accurate records of interviews conducted and consent were kept under lock and key to facilitate the audit trail. The services of a co-coder were used to promote conformability.

Transferability: The study setting was described, and research methodology and data collection and analysis procedures were explained in detail in order to allow other researchers to determine the transferability of the research findings to their own context.

Authenticity: peer review and member checks were implemented to ensure the authenticity of the data.

## Results

### Participant’s profile

Two hundred and twelve professional nurses were recruited for the study. Fifty-nine per cent of the participants had a diploma in nursing followed by 20% with a BCur, 17% with an advanced diploma in nursing and 1% with a master’s degree in nursing. Most of the participants worked in intensive care units followed by trauma and emergency, general wards and management, respectively. The majority of the participants had more than 5 years’ experience in nursing; the minority were on the night shift while the majority were on the day shift. Most of the study participants were satisfied with their jobs. The acronyms P and S are employed to distinguish between the responses obtained from the surveyed hospitals.

The main theme was contributory factors related to patient safety incidences.

As listed in Table 1, the five sub-themes from the data analysis, namely, poor understanding of the concept of patient safety, non-adherence to patient safety protocols, inadequate management support for patient safety incidents, ineffective communication and feedback and safety audits.

**TABLE 1:** Main themes and sub-theme.

Main theme	Sub-theme
Contributory factors related to patient safety incidences	Poor understanding of the concept of patient safety
	Non-adherence to patient safety protocols
	Inadequate management support for patient safety incidents
	Ineffective communication and feedback
	Safety audits

### Sub theme 1: Poor understanding of the concept of patient safety

Participants were required to choose from a series of options, what they understood as patient safety. Most of the respondents demonstrated their understanding of patient safety as the prevention of falls, medication errors and prevention of patient self-extubation, and this demonstrated a poor understanding of the concept patient safety:

‘Patient safety is the prevention of falls.’ (S34, P2)

‘Patient safety is the prevention of patient self extubation and medication errors.’ (P6)

‘Patient Safety is the prevention of patient falls and medication errors.’ (P44)

‘Patient safety is the prevention of medication error.’ (S13)

Subsequent questions asked to explore the reasons for the misunderstanding of the concept patient safety revealed that there were no patient safety champions designated to oversee patient safety issues and incidents through the provision of regular teachings, in-service training and guidance to the nurses:

‘We do not have patient safety champions.’ (P16, P5)

‘There is no designated nursing staff allocated to oversee patient safety related issues.’ (S17, S24)

The study participants mentioned that to improve nurses’ knowledge on patient safety incidents, continuous development education must be provided in the form of in-service training to nurses on regular basis. The education of staff on patient safety should be promoted through continuous professional development programmes:

‘The various training institutions must emphasise patient safety during the period of training of the nurses in order to instil patient safety practices in the upcoming generation of nurses.’ (S96, P77)

‘Nurses must know and understand the patient safety policies in place and monthly in-service training should be conducted to provide nurses with in-depth knowledge on patient safety issues.’ (S46, P69)

### Sub-theme 2: Non-adherence to patient safety protocols

Health care settings are required to have patient safety protocols in place to improve patient outcomes and reduce patient safety incidences. The researcher explored the ability of nurses to adhere to these protocols. Most of the study participants confirmed that they are unable to comply with the checklist in place for patient safety because of the following reasons: minimum time, excessive number of patients allocated to be nursed, too much paperwork and tiredness because of long working hours, excessive workload and unavailability of resources. With regard to minimum time and too much paperwork, participants had this to say:

‘The documentation of service rendered is important for continuity of care and also for legal purposes, however, documentation is cumbersome such that the hour’s required spent on documentation exceeds the hours put into patient care.’ (S19, P63)

The unavailability of resources for use by the nurses during direct patient care also contributed to poor adherence to protocols with regard to the use of personal protective clothing. Participants stated the following:

‘There are no PPEs readily available by the bedside for easy access and use.’ (P16)

Also, the nurse–patient ratio in terms of the patients allocated to each nurse for wholly compensatory care has been increasing. Participants described it like this:

‘Critically ill patients who require intensive care [*ratio of 1:1*] are doubled up putting a strain on the nurse.’ (P19)

Staff shortage came out as an issue of concern as the majority of the study participants reported inadequate staffing as one of the main contributory factors to poor patient care:

‘A staff nurse ratio of 1 nurse: 8 to 12 patients place a lot of strain on the nurse (burnout) and, thus, render her incapable of providing holistic and quality care to the patients.’ (S14)

‘A critically ill patient may require two nurses at a time however this is not considered due to staff shortages.’ (S10)

‘The issue of staff shortages is huge concern that must be addressed as adequate staffing is required to maintain quality patient care and ensure the patient is safe at all times.’ (P54)

‘Some nurses neglect the alarm settings and sometimes ignore the alarm when it rings due to increased work load, and this almost costs a patient his life.’ (S23, P5)

The study participants highlighted the importance of teamwork, stress management and addressing staff shortages in the nursing units as a means to promote patient safety. Study participants mentioned the need for nurses to work as a team, support each other with regard to the prioritisation of the safety of the patients under their care:

‘Patient safety must be a shared responsibility among staff and between patients however there is no unity amongst staff. Nursing staff are very selfish and do not want to support each other in our line of duty.’ (P47, S96)

Participants confirmed their inability to adhere to all safety protocols in place because of various reasons; however, they agreed that the reasons could be overcome with teamwork and support:

‘Through collaboration and team efforts, we can deliver quality health care to improve patient outcomes.’ (P49)

### Sub-theme 3: Inadequate management support for patient safety incidents

Participants mentioned that the ill provision of patient safety tool kits by the management contributed to poor nurses’ compliance to patient safety needs:

‘Protocols and policies are not clearly displayed by the bedside of each patient hence difficulty with adherence.’ (S98, P25)

Participants also mentioned the unavailability and accessibility of standardised operative protocols on patient safety incidents cause inconsistencies and distortion in the rendering of safe nursing care:

‘Available quality alerts are not specific to the units; they are generalised hence may not be applicable to all units. The unit must write up quality alerts based on frequent patient safety incidents and tailored to suit the unit.’ (P88, S19)

Recommendations from the study participants indicated that management must ensure that the private health institutions introduce the following management tools for patient safety, namely, quality alerts, surveillance and knowledge acquisition; as they acknowledged that these measures would inform staff of risky behaviours and unsafe practices that expose patients to harm.

With regard to quality alerts, participant mentioned the following:

‘Checklist in use for patient safety must be the same for each unit in line with the National Safety Guidelines and should not vary between units.’ (P55, S33)

To improve patient safety practices based on surveillance participants had this to say:

‘Spot checks are not frequently done; hence mistakes or errors go unnoticed. The occupational health nurse should conduct spot checks to detect any risky behaviour early that may put the safety of patients in jeopardy.’ (P87)

‘A safety champion must be designated to conduct frequent patient safety checks to assess the nurses’ compliance with the protocols in place.’ (S27)

Participants perceived that regular training aimed at improving nurses’ knowledge on patient safety-related issues will help improve positive patient care outcome. Participants stated the following:

‘Management should support staff through the provision of regular in-service training.’ (S44)

‘Acquisition of knowledge on patient safety issues, will empower the nurse to deliver adequate care.’ (P72)

### Sub-theme 4: Ineffective communication and feedback

Majority of the study participants attributed patient safety incidents to poor communication channels within the organisational structure and among multi-disciplinary staff:

‘There are no clear and open communication channels between hospital management and nurses to allow nurses to express the challenges and circumstances impacting on patient safety in order to ensure good rapport and to promote the delivery of safe care.’ (S24)

‘There is no proper information shared between physiotherapist, nursing staff and attending patient specialist on the focus areas and treatment outcomes for patients.’ (P23)

‘Attending doctors should discuss the patient’s prognosis and the specific medical needs of the patient clearly with the nursing personnel to ensure safe care.’ (S20)

‘Clear communication of updated patient safety measures and policies available globally are not made readily available to staff hence we are left to make assumptions.’ (P33)

### Sub-theme 5: Safety audit

The participants highlighted the importance of safety audits being conducted on a regular basis as a means of evaluating care given and also providing feedback to staff with regard to patient care:

‘Safety audits are only carried out after discharge thus staff inadequacies are not picked up early for rectification. Safety audits must be conducted while the patient is in hospital and not only after discharge to enable the nurse to evaluate to what extent the patient is satisfied with the care, he/she is receiving, and also to adopt changes if necessary.’ (S77)

Absence of regular safety audits and real time feedback coupled with good communication channels was identified as a contributory factor to poor patient care outcomes.

## Discussion

Contributory factors in the context of this study were seen as activities or practices that may compromise quality patient safety outcomes. The contributory factors linked to poor patient safety practices that emerged from the study included the following: lack of knowledge on patient safety, unavailability of patient safety champions, lack of in-service training on patient safety issues, staff shortages, inadequate nurse–patient ratio, burnout, poor communication channels that persist in the healthcare environment and safety audits.

The study revealed that the majority of professional nurses lacked adequate knowledge on patient safety as they limited patient safety incidents to falls, medication errors and self-extubation. In contrast to studies conducted by Murray, Sounding and Cope (2019:2547) in which all the nurse participants were unanimously found to fully understand the concept of patient safety, the findings of this study are similar to those of a study conducted by Biresaw, Asfaw and Zewdu (2020) who assessed healthcare professionals’ knowledge of patient safety; their study found that 51.6% of the nurses’ (respondents) knowledge of patient safety was poor. Similarly, a study by Brasaite et al. (2017) in Ethiopia on the nurses’ knowledge of and attitudes towards patient safety and its associated factors also revealed the respondents’ knowledge of patient safety to be generally poor.

A majority of the study participants indicated that there were no safety champions in their unit. Patient safety champions are persons who are specifically designated to vigorously support patient safety, thereby improving quality patient outcomes (Tolentino et al. 2018). As found in the study conducted by Hurtado et al. (2020), the use of safety champions results in a significant improvement in patient safety outcomes. The absence of a patient safety champion implies that there are no designated personnel members who supervise and ensure the implementation of patient safety policies to promote quality patient care.

The study revealed that although there were policies in place for patient safety, it was not possible for most of the study participants to comply with these policies. Non-compliance with patient safety regulations and policies is known to be associated with poor patient outcomes. These were the findings in studies conducted by Da Silva Gama et al. (2019) and Vaismoradi et al. (2020). This result explains why patient safety issues persist and remain an issue of concern.

The data gathered from the study participants implied a lack of regular in-service training for nurses. Regular in-service training is expected to improve the nurses’ knowledge and provide them with updated information on patient safety issues and statistical effects of patient safety incidents (Amiri, Khademian & Nikandish 2018; Kim et al. 2019; Wagner, Dolansky & Englander 2018). It was concluded from the result pertaining to in-service training that the nurses were not receiving the required updates and information on patient safety, and, consequently, the likelihood was that they would not appreciate patient safety as an issue of concern and its important role in quality improvement outcomes. Accordingly, they would not appreciate the need to adhere to safety protocols. Periodic in-service training is essential to increase the existing practical knowledge base of nurses (Vaismoradi et al. 2020), improve and maintain high-quality care and nursing standards (Kim et al. 2019) and thus improve patient care satisfaction outcomes.

Nursing staff shortages is an issue of increasing concern. The responses from the study participants indicated the issue of staff shortages, which resulted in low nurse–patient ratios and increased workload as contributory factors to poor patient safety outcomes. It has been found that every extra patient on a nurse’s case load increases the mortality rate by 7% (Khoshakhlagh et al. 2019). Staff shortages affect areas such as patient and family education and engagement in the care process, routine environmental checks to assess any dangers promptly, good and clear communication and report writing, appropriate physical assessment of patients, frequent shift leader rounds and the implementation of infection control practices. Nurse shortages are also known to cause errors that result in higher morbidity and mortality rates (Haddad, Annamaraju & Toney-Butler 2020). Burnout resulting from excessive workloads, long journeys and ineffective interpersonal relationships is associated with worsening patient safety and poor quality outcomes, whereas a good working environment, the safe staffing of nurses, and in-service education are associated with decreased hospital lengths of stay, the decreased incidence of patient harm and infections and an overall decrease in mortality (Kheswa 2019; Lee et al. 2021; Mersin et al. 2020; WHO 2019). Nurse burnout is characterised by a reduction in energy levels, emotional and physical exhaustion, a lack of motivation and feelings of frustration, often leading to a decrease in work efficiency (Mudallal, Othman & Al Hassan 2017). Burnout is often a feature of the nursing profession, and the professional nurse is prone to experience it because of the nature of the work. In addition, burnout may impact adversely on the nurse’s health, compromising her ability to deliver the quality of care required and, thereby, compromising patient care and quality safety outcomes (Rachel & Francesco 2018).

Most of the study participants acknowledged the fact that unit safety protocols and checklists for general and some specific procedures to be conducted by the healthcare providers were in place and available but that staff members were often non-compliant because of communication issues. Failures in communication, for example, poor handover, have been shown to account for most adverse outcomes in hospitals. Poor handovers prevent important information from being passed from one nurse to the other, thus resulting in errors, care omissions, treatment delays, inefficiencies, inappropriate treatment and increased hospital costs (Vaismoradi et al. 2020:2028). In their study to assess patient safety culture as perceived by nurses, Alquwez et al. (2018) found poor communication to be one of the weaknesses in a patient safety culture.

The data gathered suggested the occurrence of irregular patient safety audits and irregular staff performance reviews. Poor and irregular safety audits are said to be associated with poor adherence to patient safety policies. This was seen in the systematic review conducted by Vaismoradi et al. (2020) to synthesise knowledge and explore factors that influence nurses’ adherence to patient safety principles. To ensure patient safety while patients are in hospital, it is imperative that regular patient safety audits are conducted to determine patient care areas that require improvement, as well as after discharge, to rate the patient care and address all concerns and areas in need of improvement (Farokhzadian, Nayeri & Borhani 2018). The lack of regular staff performance reviews on patient safety to appraise nurses who are compliant with safety protocols and to implement corrective actions in respect of those nurses who are struggling to adhere to safety protocols impedes reviewing and addressing staff needs or challenges regarding patient safety issues and quality outcomes (Heldal, Kongsvik & Håland 2019). In their study, Pelzang and Hutchinson (2018) found clinical governance, which includes safety audits and regular feedback, to be an effective strategy to ensure and improve quality patient safety outcomes.

### Study recommendations

Unit managers must designate patient safety champions to ensure support for and compliance with patient safety protocols; facilitate teamwork by organising structured team-building activities to serve as a support system for nurses. Private hospital groups must develop and align the group patient safety policies and protocols with the National Department of Health’s Patient Safety policy; establish nurse patient safety forum to increase nurse’s awareness of patient safety to promote compliance and, thereby, improve quality patient care outcomes. Furthermore, private hospital groups should introduce mandatory patient safety training and education to enhance the skills and knowledge of professional nurses, thus ensuring quality patient outcomes.

Professional nurses must comply with written reporting requirements in relation to patient safety issues as the study found communication to be one of the main areas of concern. Professional nurses can keep journals to guide their action plan (write in to-do list) for the patients in their care. It is vital that nurses are encouraged to report for duty timeously to ensure accurate handovers and report taking. Patient progress reports must be written in a clear and legible handwriting for effective continuity of care. Nurses must adhere to the organisation’s main medium of communication (usually English) when writing reports or handing over verbally to the nurses taking over patient care from them.

We recommend patient safety compliance review and evaluation of nurses’ knowledge and practices in respect of patient safety issues. This can be achieved through peer reviews and assessment done at the end of each shift, to identify any inadequacies and risky behaviours. This will enable prompt and corrective action to be taken to control the rate of occurrence of patient safety incidents.

### Strengths

The study utilised a qualitative approach that allowed the issue under study to be examined in details and in depth. Results of this study have brought to light the challenges related to patient safety from the nursing perspective. Accordingly, the results of the study and recommendations may be used to create awareness on patient safety and develop healthcare compliance guidelines for patient safety education.

### Limitations

The study focused on only two private healthcare facilities out of the many in the country. Thus findings cannot be transferable to other public health facilities.

## Conclusion

This study thoroughly investigated factors contributing to patient harm and injury in a hospital environment. The contributory factors linked to poor patient safety practices that emerged from the study included lack of knowledge on patient safety, unavailability of patient safety champions and lack of in-service training on patient safety issues, staff shortages, inadequate nurse patient ratio and burnout, poor communication channels that persist in the healthcare environment and irregular safety audits. Educating nurses on patient safety issues and incident report writing, creating awareness through frequent unit meetings, providing nurses with the required support and assistance, implementing safety champions for each unit, and capacity building will help to ensure that nurses adhere to patient safety protocols, thereby improving patient safety quality outcomes.
